# The Shape Effect of Acoustic Micropillar Array Chips in Flexible Label-Free Separation of Cancer Cells

**DOI:** 10.3390/mi15040421

**Published:** 2024-03-22

**Authors:** Lin Lin, Rongxing Zhu, Wang Li, Guoqiang Dong, Hui You

**Affiliations:** 1 Key Laboratory of Disaster Prevention and Structural Safety of Ministry of Education, Guangxi University, Nanning 530004, China; q1329728621@163.com (R.Z.); 18692045737@163.com (W.L.); guoqiang20000221@163.com (G.D.); 2 School of Mechanical Engineering, Guangxi University, Nanning 530004, China; 3 Guangxi Key Lab of Manufacturing System and Advanced Manufacturing Technology, Nanning 530003, China

**Keywords:** circulating tumor cells, micropillar array, acoustic streaming, microfluidics, size separation

## Abstract

The precise isolation of circulating tumor cells (CTCs) from blood samples is a potent tool for cancer diagnosis and clinical prognosis. However, CTCs are present in extremely low quantities in the bloodstream, posing a significant challenge to their isolation. In this study, we propose a non-contact acoustic micropillar array (AMPA) chip based on acoustic streaming for the flexible, label-free capture of cancer cells. Three shapes of micropillar array chips (circular, rhombus, and square) were fabricated. The acoustic streaming characteristics generated by the vibration of microstructures of different shapes are studied in depth by combining simulation and experiment. The critical parameters (voltage and flow rate) of the device were systematically investigated using microparticle experiments to optimize capture performance. Subsequently, the capture efficiencies of the three micropillar structures were experimentally evaluated using mouse whole blood samples containing cancer cells. The experimental results revealed that the rhombus microstructure was selected as the optimal shape, demonstrating high capture efficiency (93%) and cell activity (96%). Moreover, the reversibility of the acoustic streaming was harnessed for the flexible release and capture of cancer cells, facilitating optical detection and analysis. This work holds promise for applications in monitoring cancer metastasis, bio-detection, and beyond.

## 1. Introduction

Malignant tumors are one of the major diseases seriously threatening human health and life, with an extremely high fatality rate [1]. Approximately 90% of cancer-related deaths are caused by tumor metastasis, making early detection and treatment a critical means of preventing death [2,3]. In the process of tumor metastasis, circulating tumor cells (CTCs) detach from the primary tumor and enter the peripheral blood, traveling through the bloodstream to other organs and tissues in the body, ultimately forming new tumor metastases. As “seeds” enter the peripheral blood circulation, circulating tumor cells contain crucial information about the origin, development, and migration of tumors [4,5]. Therefore, isolating CTCs from the blood of cancer patients, monitoring their types, and tracking changes in their quantity can facilitate real-time monitoring of tumor metastasis, assessment of treatment effectiveness, and the development of personalized treatment plans [6,7]. However, the number of CTCs in the blood of cancer patients is much lower than that of other blood cells [8] (blood cells: CTCs = 10^9^:1), and blood cells interfere with the precise capture of CTCs, which greatly limits the efficiency of CTCs capture. Therefore, the development of a precise, simple, and biocompatible CTC isolation technique is necessary to overcome this great challenge.

Microfluidic chips have become an ideal platform for the isolation of CTCs from blood samples by virtue of their low consumption, miniaturization, rapid analysis, and easy integration [9]. Currently, microfluidics-based methods for the isolation of CTCs can be classified into affinity and physical methods. Affinity-based methods rely on specific binding between antibodies or magnetic beads and unique markers (such as EpCAM) on the surface of circulating tumor cells to achieve CTC separation [10,11,12]. Based on this principle, some technologies for capturing circulating tumor cells have been commercialized, such as the Food and Drug Administration (FDA)-approved CellSearch^®^ system [13,14]. However, this system suffers from high cost, poor specificity, and low separation efficiency in practical operation, which limits its clinical application [15,16]. Physical methods utilize physical fields like acoustic, electric, or magnetic fields [17,18,19,20,21] as well as filtration and hydrodynamic methods [22,23,24] to separate CTCs from blood based on their physical characteristics (such as size, density, dielectric properties, deformability). Electrical separation methods utilize the different dielectric properties of cancer cells and blood cells in an electric field, which are driven to deflect the cancer cells in different directions for the purpose of separating the cancer cells. This method uses electric field regulation, which is prone to generate high joule heat, affecting cell activity and not conducive to the subsequent culture and detection of cancer cells. In addition, the manipulation process is cumbersome, and it involves the preparation of the conductivity solution and the regulation of the electric signal. Magnetic separation methods often require pre-treatment of the blood sample first, to which functionalized magnetic beads are added to bind specifically to the target cancer cells. By adjusting the strength and direction of the external magnetic field, the target cancer cells can be effectively separated from the mixture. This method requires specific labeling, and the separation efficiency is limited by the degree of binding of the cancer cells to the magnetic beads. In addition, the formulation of functionalized magnetic beads will increase the complexity of manipulation and the cost of separation. The filtration separation method realizes the separation of cancer cells by reasonably setting the parameters, such as the spacing of microcolumns as well as the pore size of the film, according to the differences in size and deformability between cancer cells and blood cells. However, its special design principle and operation method also bring a lot of limitations, such as low flux during the separation process, ease of clogging, low separation efficiency due to the uneven size of the cell community, ease of inactivating the cells, and so on. The hydrodynamic separation method is to make changes in the trajectory of the cells by setting up a barrier or controlling the fluid contraction and expansion in the channel and utilizing the differences in size and density between the cells to cause them to be subjected to different fluid forces. This method has high flow rates that can affect cell activity, and the fixed design of the microchannels and structures limits the ability to process different samples. In comparison to affinity methods, physical techniques do not depend on specific markers. They are characterized by simplicity and efficiency in operation, holding greater potential for advancing cancer cell capture techniques. Among various label-free physical methods, acoustic methods stand out for their good biocompatibility, flexible manipulation, and low energy consumption, making them an ideal choice for developing microfluidic chips for isolating CTCs.

Acoustic separation technology is primarily categorized into two types: surface acoustic waves (SAWs) [25,26] and bulk acoustic waves (BAWs) [27,28]. SAW devices (operating at frequencies of 1–1000 MHz [20]) generate a high-intensity acoustic field and utilize the powerful force of acoustic radiation to promote the migration of cancer cells toward the acoustic nodes, thus enabling the isolation of cancer cells [29,30]. However, this approach requires the introduction of sheath flow focusing before the cells reach the acoustic manipulation region, which may contaminate the sample as well as affect cell activity. Compared to SAW methods, BAW devices(operating frequencies in the 1–1000 KHz [31]) are often combined with bubbles or solid microstructures to achieve versatile and efficient sample manipulation capabilities at lower fabrication costs as well as power requirements. Wang et al. [32] achieved selective capture, accumulation, and release of particles depending on the size difference by combining a stabilized background flow with a microbubble acoustic microfluidic technique. Tang et al. [33] employed a strategy of parallel capture and rotation of cells by utilizing the acoustic streaming generated by an array of regularly arranged bubbles in a vibrating microchannel. Jiang et al. [34] utilized side-chambered microbubble acoustic microfluidics to achieve high-throughput separation of CTCs from the blood of clinical cancer patients with guaranteed functional integrity of the cells. Although bubble-driven acoustic systems are effective in cell separation, they are often limited by the inconvenience of bubble capture and the instability of the bubble structure during operation. Compared with microbubble-based chips, acoustic-driven microstructures (micropillars [35,36,37], microcavities [38,39], and sharp edges [40,41]) have emerged as a promising non-contact separation technique due to their maneuvering stability, lack of need for sheath flow, and strong capture power. Additionally, the power and frequency requirements for this method are significantly lower than those used in medical ultrasound imaging, ensuring minimal impact on cell viability and functionality [42,43].However, in microfluidic systems, the shape of microstructures in the channels is a critical design variable influencing fluid distribution. Sharp edges enable the rotation of HeLa cells up to 1400 rpm [40], while asymmetrical sharp edges induce the rotation of Diatom cells up to 1800 rpm [44]. Moreover, Meng et al. [45] demonstrated controlled transport of particles and cells by designing and arranging microcolumn shapes appropriately. The individual above studies have shown that acoustically driven microstructures exhibit the potential to capture cancer cells in parallel from the bloodstream, but the effect of the shape of the microstructures on the separation of cancer cells has not been reported.

In this study, we analyzed the effect of the shape of the micropillars on the separation efficiency of cancer cells to maximize the separation of CTCs. We propose an acoustic micropillar array chip whose mechanism of operation relies on the cell size effect, utilizing the acoustic streaming effect and acoustic radiation force generated by vibrating micropillar arrays for separation. This device introduces an array of micropillars into the microchannel, not only possessing the capability to handle large cell samples but also expanding the spatial manipulation of cells. In comparison to previous acoustic separation devices, this strategy eliminates the need to introduce sheath flow focusing, offering advantages such as simplicity in manufacturing and ease of integration. In this work, a variety of micropillar structures, including circular, square, and rhombus, were used, and the size and distribution of the acoustic streaming generated by the vibration of the microstructures were modeled by simulation, which was in high agreement with the experimental results. Subsequently, the capture capability of the device was validated via experiments using polystyrene microspheres, and key parameters (voltage, flow rate) of the entire system were optimized. Finally, breast cancer cells were used as a representative of CTCs, which were added to mouse blood samples (1:4), and the capture efficiencies of the three micropillar structures were further evaluated by the results of separation experiments. The proposed AMPA chip in this study demonstrates effectiveness and flexibility in isolating cancer cells, making a significant contribution to clinical applications.

## 2. Materials and Methods

### 2.1. Composition and Working Principle of AMPA Chip

**Figure 1:**
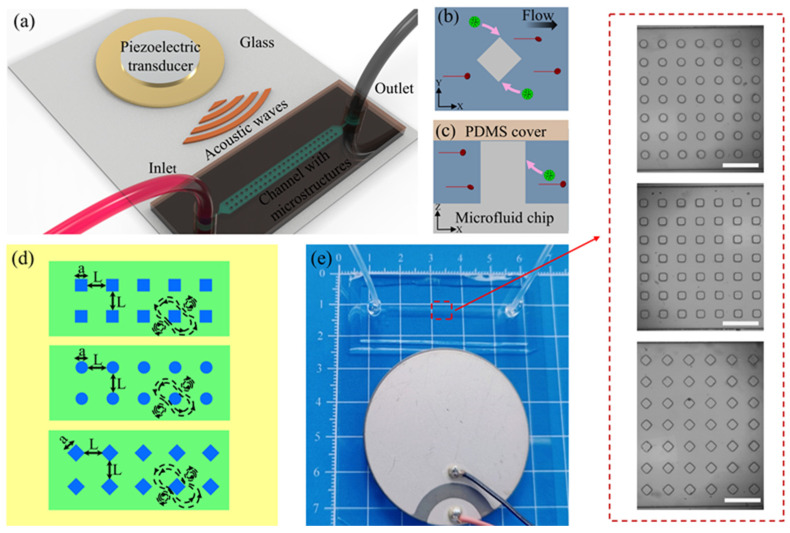
Schematic diagram of an AMPA chip based on an oscillating micropillar array for cell separation. (**a**) General schematic of the AMPA chip. The process of capturing and separating cancer cells using acoustic streaming generated by microstructure vibration: (**b**) top view and (**c**) side view. (**d**) Top view of the microfluidic chip fixed on a slide (yellow). The microstructural arrays consist of square, circular, and rhombus micropillar structures (blue, with feature size a and micropillar spacing L) and main channels (green), respectively. (**e**) Physical view of the device, the inset shows images of three different types of microstructures arranged in patterns in the main channel. Scale bar: 500 µm.

The AMPA chip (Figure 1a) is composed of a polydimethylsiloxane (PDMS) microfluidic channel, a PDMS cover for channel sealing, a piezoelectric transducer for generating ultrasound (US), and a thin glass substrate. The PDMS channel includes an array of micropillar structures, with an inlet for loading initial mixed biological samples and periodic washing and an outlet for collecting samples at the end of the experiment.

Figure 1b,c show a schematic diagram of the device capturing and isolating cancer cells. When the US excites the microstructures, acoustic streaming is induced in the surrounding fluid, generating a powerful acoustic trapping force that is utilized for the capture of target cancer cells (green), while smaller red blood (red) cells are carried away by a stable background flow. Three micropillar structures (circular, square, and rhombus) were used to evaluate the effect on the separation efficiency of cancer cells. Figure 1d presents a top view of the microfluidic chip, including three channels with different microstructure arrays and their corresponding acoustic streaming patterns. Considering the chip printing accuracy as well as the convenience of comparative analysis, the feature size of the final microfluidic chip microstructures a=100 µm and the spacing between microstructures L=100 µm. In addition, the channel height is designed to be approximately equal to the height of the microstructures to prevent the escape of target cancer cells from above. The physical diagram of the AMPA chip is shown in Figure 1e. There are 120 × 7 micropillar structures in the microchannel, which are evenly distributed in the working area. A single microstructure can capture two cancer cells, and the maximum capture limit of this design of microfluidic chip is 1680. The inset in Figure 1e shows a magnified view of three different microstructures in the microchannel.

When suspended particles are in an acoustic streaming field, the time-averaged acoustic force they experience includes both the acoustic radiation force $F_{rad}$ (generated by the scattering of sound waves on the particles) and the Stokes drag force $F_{d}$ (arising from the acoustic streaming). When the radius of spherical particles is much smaller than the wavelength of the sound wave (a≪λ),acoustic radiation force can be calculated via the gradient of the Gor’kov potential U as [46]:

(1) $$F_{rad}=-\nabla U$$

(2) $$U=2\pi a^{3}\left[\frac{f_{1}\kappa_{0}}{3}\langle p_{1}^{2}\rangle -\frac{f_{2}\rho_{0}}{2}\langle v_{1}^{2}\rangle \right]$$

(3) $$f_{1}=1-\frac{\kappa_{p}}{\kappa_{0}},f_{2}=\frac{2\left(\rho_{p}-\rho_{0}\right)}{2\rho_{p}+\rho_{0}}$$

where a is the radius of the particle, $\langle p_{1}^{2}\rangle$ and $\langle v_{1}^{2}\rangle$ are the mean square fluctuations of the ultrasonic standing wave pressure and velocity, respectively, $\rho_{p}$ is the density of the particle, $\rho_{0}$ is the density of the fluid, *κ_p_* is the particle compressibility, $\kappa_{0}$ is the fluid compressibility.

Stokes drag force on the particles is calculated from the following expression:

(4) $$F_{d}=6\pi a\eta \left(\langle v_{2}\rangle -u\right)$$

where η is the dynamic viscosity of the fluid, $\langle v_{2}\rangle$ is the time-averaged acoustic velocity, and u is the velocity of the particles. From Equations (2) and (4), it can be inferred that the acoustic radiation force and Stokes resistance acting on the particles are positively correlated with the particle diameter. Therefore, at the same acoustic vibration, particles with larger diameters will be subjected to a larger acoustic capture force (compared to particles with smaller diameters) and will be more easily captured.

### 2.2. AMPA Chip Fabriction

**Figure 2:**
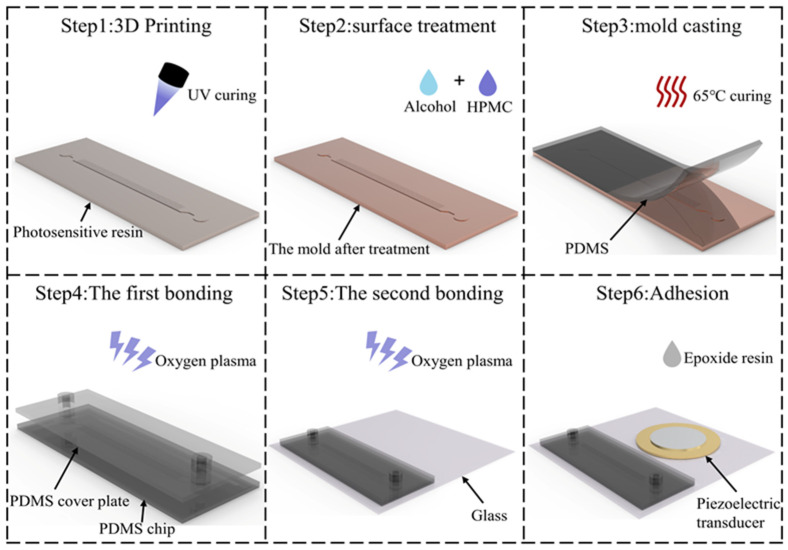
Fabrication Process of AMPA Chip.

We fabricated a PDMS microfluidic chip for cell separation using a soft lithography process, as shown in Figure 2. The specific steps are as follows:

- 3D printing: A high-precision 3D printer (NanoArch P140, Shenzhen MoFang New Material Technology Co., Ltd., Shenzhen, China) was used to rapidly fabricate the master molds.
- Surface Treatment: To improve the hydrophilicity of the chip surface, the chip mold was magnetically stirred (1000 rpm) in the diluted aqueous solution of the hydrophilic polymer hydroxypropylmethylcellulose (HPMC) for 1.5 h and dried with nitrogen. The solution was configured according to previous research [47].
- Mold Casting: Sylgard 184 silicone elastomer curing agent (Dow Corning, Midland, MI, USA) is mixed with Sylgard 184 Silicone elastomer base at a 1:10 mass ratio. The mixture was poured onto a hydrophilic-treated master mold, degassed under vacuum for 5 min, and then baked in a vacuum drying oven (DZF-6090, Shanghai Jinghong Experimental Equipment Co., Ltd., Shanghai, China) at 65 °C for 5 h. The cured PDMS peeled from the master mold.
- First Bonding: The PDMS microfluidic chip and the punched PDMS cover plate (2 mm in diameter) were processed in a plasma cleaner (PDC-002, Harrick Plasma, Ithaca, NY, USA) for 5 min and firmly bonded.
- Second Bonding: The bonded microfluidic chip and glass substrate are plasma-treated for 5 min, are firmly bonded and then heated at 85 °C for 1 h to ensure bond stability.
- Adhesion: A piezoelectric transducer (PZT4, Zhuhai Jiaming Electronic Technology Co., Ltd., Zhuhai, Guangdong, China) has adhered to the same glass substrate using epoxy resin (Deli, Deli Group Co., Ltd., Ningbo, Zhejiang, China).

### 2.3. Sample Preparation

A suspension of monodisperse polystyrene microspheres with diameters of 2, 5, 10, 20, and 30 µm (5 mL, 1 wt%, Shanghai Yiyuan Biotechnology Co., Ltd., Shanghai, China) was used for the optimization of the acoustofluidic manipulation system parameters (voltage, flow rate) in the experiments. For fluorescence experiments, 2 µm monodisperse fluorescence functionalized microspheres (0.5 mL, 2.5 wt%, Shanghai Yiyuan Biotechnology Co., Ltd., Shanghai, China) were used to characterize the acoustic streaming generated by microstructure induction. Adding Tween 20 (BioFroxx, Beijing, China) with a concentration of 0.3% *v*/*v* prevents particles from aggregating and sticking to the channel.

Human breast cancer cells (MDA-MB-231) were purchased from the National Experimental Cell Resource Sharing Platform. Cancer cells were cultured in DMEM medium (Gibco, Carlsbad, CA, USA) supplemented with 10% fetal bovine serum (Gibco, CA, USA), 1% penicillin-streptomycin (PS) (Solarbio, Beijing, China), and 1% glutamine in the 5% CO_2_ incubator at 37 °C. Prior to the experiment, the cancer cells were diluted to a concentration of 2 × 10^5^ cells/mL and washed twice with PBS (Solarbio, Beijing, China) to remove the original residual serum. Reagent 1, the above concentration of cancer cell solution was configured with a blood sample diluted in a certain ratio (1:50) for clear photography of the cancer cell capture process. Reagent 2, 100 cancer cells were added to 1 mL of diluted (1:4) blood for subsequent cancer cell sorting experiments.

### 2.4. Experimental Setup

**Figure 3:**
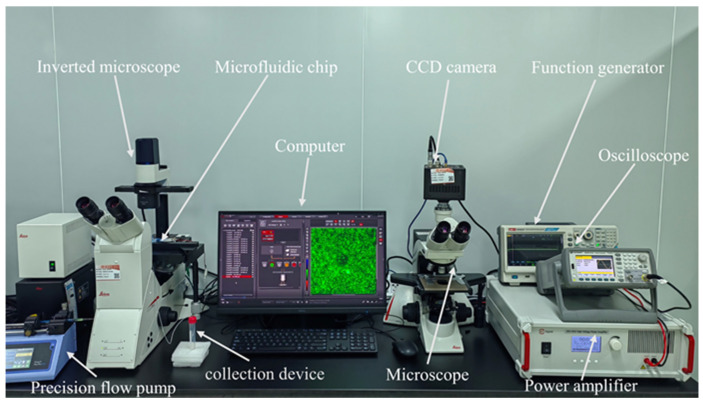
Design of the experimental platform.

Figure 3 shows the detailed setup of the acoustofluidic manipulation platform A function signal generator (33500B, Keysight, Santa Rosa, CA, USA) provided a stable periodic sinusoidal alternating current signal, which was amplified by a power amplifier (ATA-4315, Agitek, Xi’an, China) to increase the input power of the piezoelectric transducer. Meanwhile, an oscilloscope (UPO3204CS, UNI-T, Dongguan, Guangdong, China) monitored the voltage and frequency changes in real time during the experiment. The microfluidic chip was stably fixed on the microscope stage. Before running the chip, anhydrous ethanol was introduced into the microfluidic chip through the channel inlet to improve the hydrophilicity of the channel. To avoid the effect of residual alcohol on the experiment, the PBS solution was then rinsed for 5 min. Subsequently, a precision flow pump (pump 11 Pico Plus Elite, Harvard, Cambridge, MA, USA) containing the biomixed sample solution was pumped into the microchannel through a transparent rubber tube (0.7 mm × 2 mm). A fluorescence inverted microscope (DMi8, Laica, Wetzlar, Germany) was used for visual characterization of the acoustic streaming patterns generated by microstructure induction. A charge-coupled device (CCD) camera (FASTCAM MINI UX100, Photron, Nagoya, Japan) attached to the optical microscope (DM2000, Laica, Germany) was used to track the cancer cells and polystyrene particle capture process, respectively, and record the whole separation process. At the end of the particle and cancer cell capture experiment, the US device was kept on, and the microscope was moved to measure the number of all cancer cells or particles captured by the micropillar. Based on the particle solution collected at the outlet, the total number of particles entering the microchannel can be obtained by a blood cell counter (Automatic Cell Counter, C100, Reward Life Technology Co. Ltd., Shenzhen, China). The total number of cancer cells is the number passed added into the blood sample. The collection device consisted of a 15 mL plastic centrifuge tube (LAB-SELECL, Nanjing, Jiangsu, China) and a 1.5 mL microcentrifuge tube (biosharp, Nanjing, Jiangsu, China).

In order to characterize the separation performance, we employed the metric ($\eta_{c}$) of capture efficiency for cancer cells or microparticles:

(5) $$\eta_{c}=\frac{n_{m}}{n_{t}}$$

where $n_{m}$ is the number of cancer cells or particles captured by the micropillar array, and $n_{t}$ is the total number of cancer cells or microparticles passed into the microchannel.

### 2.5. Statistical Analysis

All experiments were repeated three times (n = 3), and data are expressed as mean ± SD deviation. One-way analysis of variance was used to determine the statistical significance of overall differences between multiple groups. *p* < 0.05 (*) and *p* < 0.01 (**) were considered statistically significant and highly significant, respectively.

## 3. Results

### 3.1. Study on Acoustic Streaming Induced by Vibrating Microstructures

**Figure 4:**
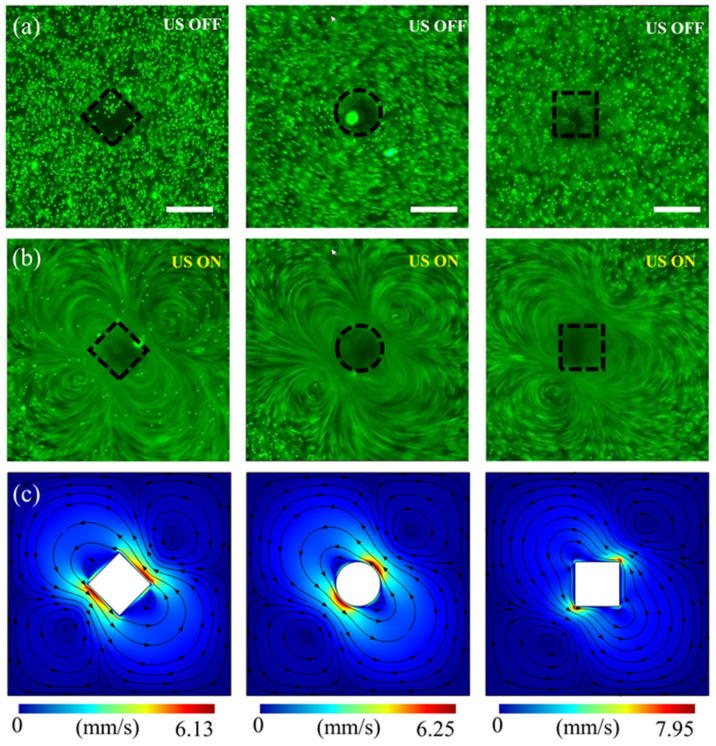
Acoustic streaming results generated by ultrasound-driven microstructures. Trajectories of fluorescent particles (2 µm) around the corresponding three microstructures (rhombus, circle, square): (**a**) US Device off and (**b**) US device on. (**c**) Numerical simulation of the acoustic streaming velocity distribution corresponding to the microstructures. The black flow lines show the shape of the acoustic streaming and the arrows represent the direction of the flow. All Scale bar: 100 µm.

The acoustic streaming generated by the vibration of a single microstructure was first investigated using the fluorescent particle tracer method and numerical simulation in the absence of background flow. It was observed in the experiment that even for the same microfluidic chip, multiple vibration modes exist at different frequencies. However, the frequency variation has a significant impact on the actual power coupled to the microstructures. Therefore, in order to ensure comparable capture results for different microstructures, the transducer needs to be driven with the same frequency. We chose to use the resonance frequency of the transducer (45 kHz) as the driving frequency. As shown in Figure 4a,b, when the US device is turned off, the fluorescent particles are randomly distributed around the microstructure. Once the US device is turned on, the acoustic vibration will be transmitted to the glass substrate, causing the microstructures to vibrate, and the randomly distributed fluorescent particles will begin to move at high speeds A primary microvortice (PM) and two secondary microvortices (SMs) are generated around the microstructure. The area covered by the PM is larger in size and is formed by particles rotating counterclockwise around the microstructure, while the SMs are located on both sides of the PM and cover a much smaller area, with particles moving in the opposite direction. Meanwhile, for the three different shapes of microstructures, the area covered by the acoustic streaming generated by the rhombus microstructure is the largest. More details about this dynamic acoustic streaming can be seen in Video S1. In addition, a finite element simulation was implemented using our previous microturbulence approach through acoustic streaming theory and simplified necessary assumptions [48,49]. The detailed procedure of the simulation are shown Appendix A.

As shown in Figure 4c, the simulation results coincide with the acoustic streaming patterns obtained from experiments, and one PM and two SMs are generated all around the micropillar. As can be seen from the distribution of the color plots, the fluid motion velocity gradient around the microstructures varies significantly, and the peak acoustic streaming velocity occurs in the region where the PM meets SMs and is close to the microstructures. It is worth noting that the acoustic streaming is larger in the relatively high-velocity region (>3 mm/s) of the rhombus microstructure as the shape of the microstructure changes. This is because, in this vibration mode, the rhombus microstructure makes the highly viscous dissipative region dispersed, resulting in a weakening of the strength of the acoustic streaming velocity and an increase in the relatively high flow velocity region. As a result, the rhombus microstructure acoustic streaming acts over a wider range, which is consistent with the experimentally observed phenomenon. However, the region of strong acoustic trapping force generated by ultrasonically vibrating the microstructure is mainly concentrated in the region close to the microstructure [45,50]. Therefore, the vibrating rhombus microstructures produce a wide range of acoustic streaming regions that are more likely to resist the background flow resistance and bring the target cancer cells into the strong acoustic capture force region. At the same time, the SMs generated by the rhombus microstructures have the shortest distance from the micropillar structure, which is closer to the strong acoustic capture force region, which also means that the cells and particles are more susceptible to capture. The above study suggests that at the same input power (frequency, voltage), rhombus microstructures have more potential to capture and separate cancer cells.

### 3.2. Optimized Parameters through Particle Capture Characterization

**Figure 5:**
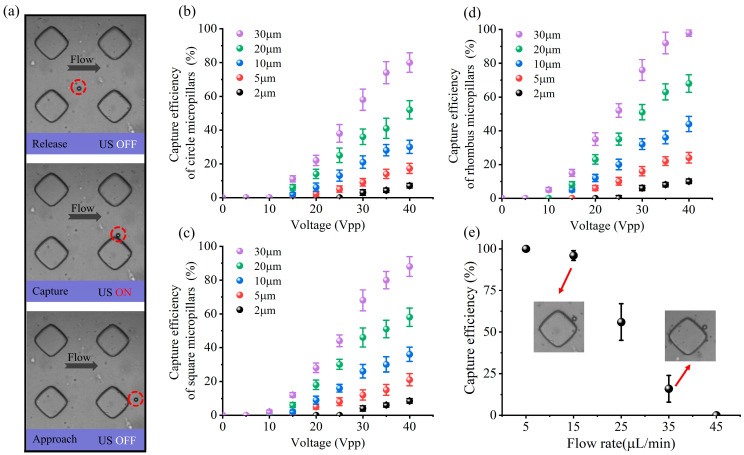
Optimization of AMPA chip parameters and investigation of capture characteristics. (**a**) Microscopic observation of the specific process of approaching, capturing, and releasing 20 µm polystyrene particles. Relationship graphs between the capture efficiency of microstructures with different diameters and driving voltage for (**b**) circular, (**c**) square, and (**d**) rhombus. (**e**) Relationship graph between flow velocity in a mixed particle solution and capture efficiency of target particles. Error bars represent standard deviation, and all experiments were repeated (n = 3).

The capability of the AMPA chip to capture microparticles was first investigated by combining a steady background flow with a controlled acoustic field. As shown in Figure 5a, when 20 µm particles are pumped into the acoustic manipulation region, they randomly pass around the microstructure. Subsequently, when the US device is turned on, the microstructure vibration generates SMs and MMs, which first pull the particles into the perimeter of the microstructure, where they are firmly captured by the acoustic trapping forces localized in the microstructure. When the acoustic trapping force generated by the vibration of the microstructure is strong enough, the target cannot be detached from the trap even when a continuous background flow is added. Only when the US device was turned off could the particles be released from the trap and flow toward the outlet (Video S2).

In order to assess the optimal drive voltage of the US device and study the influence of particle size on the capture performance of the AMPA chip, repeated experiments were conducted using polystyrene spherical particles with different diameters (2, 5, 10, 20, 30 µm). The suspension of particles with different diameters was introduced into microchannels at a flow rate of 20 µL/min. The voltage was gradually increased in steps of 5Vpp between 0 and 40 Vpp. Figure 5b–d demonstrate the dependence between the driving voltage and the capture efficiency. With the increase in driving voltage, the capture efficiency of the three microstructures for particles of all diameters increases rapidly and reaches a maximum of 40 Vpp. Simultaneously, particles with larger diameters are more easily captured because of the larger acoustic capture force on the particles. At a driving voltage of 40 Vpp, the capture efficiency of all three microstructures is higher than 80% for particles with a diameter of 30 µm. However, for smaller particles (less than 10 µm), the acoustic capture forces acting on the particles are insufficient to overcome the flow resistance, resulting in lower capture efficiency. As a result, the capture efficiency of all three microstructures for 2 µm diameter particles is less than 12%, even at a voltage of 40 Vpp. It is noteworthy that at the same driving voltage, the rhombus micropillar exhibits a higher capture efficiency for the same diameter particle compared to the other two microstructures. In particular, at a driving voltage of 40 Vpp, the rhombus micropillars show a capture efficiency of 98% for particles of 30 µm diameter, while the square micropillars show only 80%.

Under stable background flow conditions, the acoustic capture forces experienced by particles of different diameters vary. Therefore, by combining a controlled acoustic field with a steady background flow, the separation of particles of different diameters can be realized. In order to obtain the optimal background flow rate for the subsequent realization of cancer cell isolation from whole blood samples. Considering the excellent performance of rhombus micropillar structures in particle capture experiments, the device’s separation performance was tested using rhombus micropillar structures. More details on the particle separation process are shown in Video S3. As shown in Figure 5e, under the acoustic conditions of voltage of 40 Vpp, the efficiency of the micropillars to capture the target particles (20 µm) tends to decrease with the increase in the background flow rate. When the background flow rate is low (less than 10 µL/min), the capture efficiency can reach over 95%. However, as the external flow resistance increases, the acoustic capture force generated by the vibrating micropillars becomes progressively more difficult to resist. As a result, when the background flow rate reaches 25 µL/min, the capture efficiency of the microstructures for the target particles rapidly decreases to 56%. When the background flow rate is low, although the capture efficiency is high, the processing throughput of the whole device is low. We need to maximize the processing throughput of the whole device while ensuring high capture efficiency. However, the background flow rate from 25 µL/min increased the throughput of the whole device, but the particle capture efficiency was low. Therefore, we chose 15 µL/min as the optimal flow rate, which can realize high capture efficiency while maintaining good processing throughput.

### 3.3. Cancer Cell Separation

**Figure 6:**
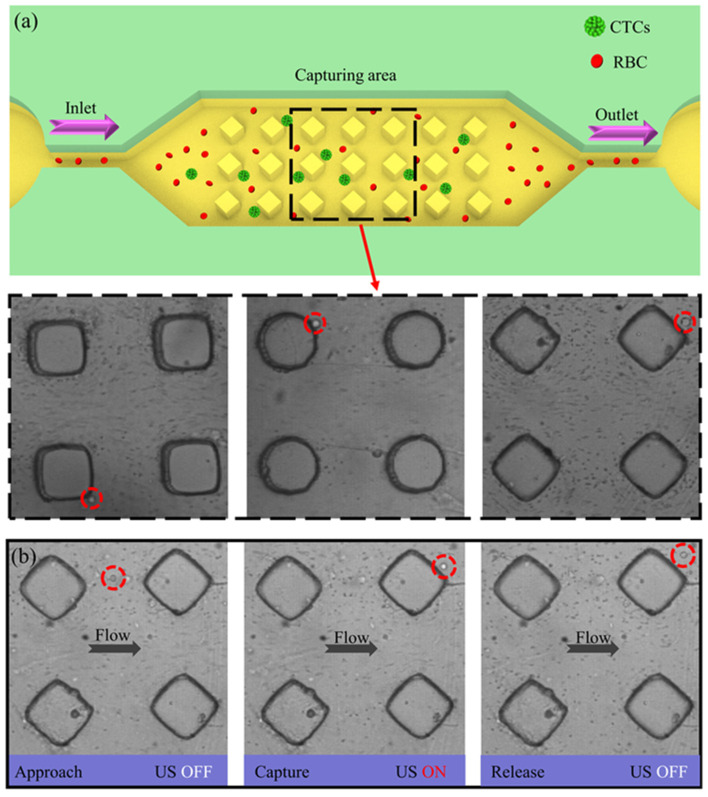
(**a**) Specific process of AMPA chip vibrating microstructures inducing acoustic streaming for parallel label-free capture of cancer cells. The inset shows that all three microstructures captured the target cancer cells. (**b**) Specific process of microscopic approach, capture and release of CTCs by acoustic rhombus micropillar structures.

After optimization of AMPA chip parameters (40 Vpp, 15 µL/min) using polystyrene particles, the device was employed for parallel label-free separation of cancer cells from mouse blood samples. Our microfluidic chip (shown in Figure 6a) is composed of three regions: (i) the inlet, where the experimental samples are injected to reach the microchannels; (ii) the capture region, which consists of micropillar structures uniformly arranged in the microchannels for precise capture of cancer cells (iii) the outlet, where the experimental samples are collected. Due to the high concentration of blood cells in untreated whole blood solution [51], it is challenging to observe the specific capture process of cancer cells. The detailed composition of the mammalian blood in question is shown in Table A2. As shown in Figure 6b and Video S4, in order to better observe the cancer cell capture process, whole blood was mixed with PBS at a dilution of 1:50, and then a portion of MDA-MB-231 cancer cells was added and stabilized, pumping into the microchannel. Turning on the US device, when the mixture reaches the acoustic capture region, the vibrating microstructure array induces the generation of SMs and MM, and cancer cells with larger diameters are captured by the strong acoustic capture force localized in the microstructures. However, the smaller diameter red and white blood cells are subjected to less acoustic trapping force, which is not sufficient to overcome the background stream flow and be carried toward the exit region.

**Figure 7:**
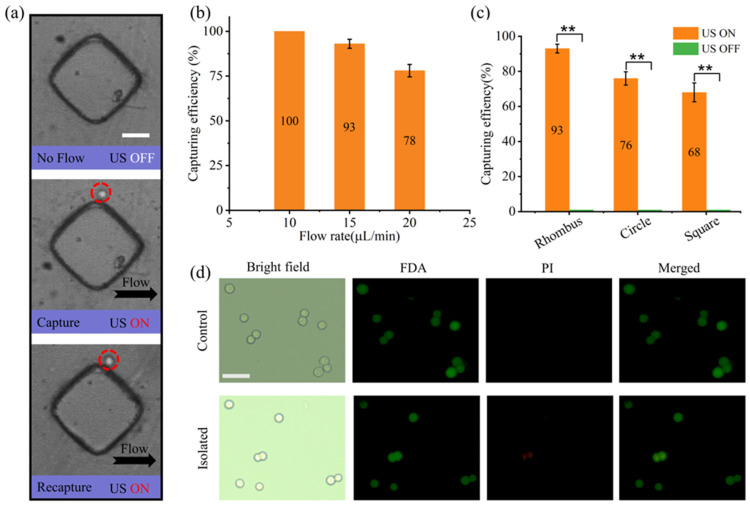
Flexibility, efficiency and biocompatibility of cancer cell capture by acoustic streaming. (**a**) The ability to flexibly capture the same cancer cells at different micropillar positions in the capture area by simply starting and stopping the ultrasound device. (**b**) Capture efficiencies of rhombus AMPA chips at flow rates of 10, 15, and 20 µL/min. (**c**) Impact of three micropillar shapes in AMPA chip on the capture efficiency of cancer cells in whole blood samples. (**d**) Bright-field and untreated cancer cell samples (upper panel) and fluorescence images of acoustically separated (lower panel). Error bars represent standard deviation, and all experiments were repeated (n = 3). All scale bar: 50 µm. *p* < 0.01 (**).

Subsequently, the flexibility of the AMPA chip in capturing and releasing target cancer cells was tested. As shown in Figure 7a, when the US device is turned on, the vibration of the micropillar causes the surrounding fluid flow, which locally generates a strong acoustic trapping force and firmly captures them in the acoustic streaming trap. Meanwhile, as long as the US device is not turned off, the trapping conditions will not change, and the cancer cells will still be firmly captured. However, the size and shape of cancer cells can vary based on diseases and other specific environmental parameters [52], and clear observation is more conducive to disease diagnosis. Therefore, by simply turning the US device on and off, we can realize different positions of the same cancer cell capture, which facilitates the observation of the structure of cancer cells. Specifically, when the US device is turned on, the cancer cells will be firmly captured in the acoustic streaming trap. Once the US device is turned off, the cancer cells are released from the acoustic streaming trap and are carried toward the exit position by the steady background flow. When the background flow rate (5 µL/min) is not significant, the US device can be turned on immediately after the cancer cells reach the vicinity of a suitable micropillar, and the cancer cells will be captured again by the acoustic streaming trap generated by the subsequent micropillar. These results indicate that this device can modulate the trapping of cancer cells at different positions in the microchannel with high flexibility.

The effect of different microstructures in the AMPA chip on the capture efficiency of cancer cells was evaluated using mouse whole blood samples. In contrast to microparticle capture experiments, whole blood samples have a more complex composition, and the sizes of MDA-MB-231 cancer cells are not uniform (13–17 µm). Therefore, we further optimized the flow rate of the system using a rhombus-shaped AMPA chip. As shown in Figure 7b, the relationship between flow rate and cancer cell capture efficiency after 5 min of complete passage of the pumping system with flow rates of 10, 15, and 20 µL/min through the capture area, respectively. The chip showed a gradual decrease in capture efficiency as the flow rate increased, and the capture efficiency decreased to 78% by applying a flow rate of 20 µL/min. To ensure high throughput with high capture efficiency, the optimal flow rate for the AMPA chip was chosen to be 15 µL/min. As shown in Figure 7c, we tested the performance of the device for cancer cell capture by pumping whole blood samples (whole blood: PBS = 1:4) into microchannels containing different microstructural array microchannels at the optimal flow rate. When the US device was turned off (control), no capture of cancer cells by the microstructures was observed. With the US device turned on, cancer cells were firmly captured by the microstructure arrays, with the rhombus-shaped microstructures showing the highest capture efficiency (93%), the circle-shaped microstructures in the middle (76%), and the square-shaped microstructures having lower capture efficiency (68%).

Finally, in order to test whether acoustic streaming has any effect on cell activity, the cell activity of collected cancer cells was assayed using fluorescein diacetate (FDA) and propidium iodide (PI). FDA is able to freely enter into living cells, but it is broken down by intracellular lipases to produce polarized, fluorescent fluorescein, causing green fluorescence in living cells. PI can only pass through the damaged cell membrane of dead cells, staining the DNA and emitting red fluorescence. Figure 7d shows the fluorescence of cancer cells collected after 5 min of treatment under sonication conditions versus cancer cells without any treatment (control). The green fluorescence represents the fluorescence of FDA, and the red fluorescence represents the fluorescence of PI. In the FDA and PI superimposed results, most of the untreated cancer cells (98%) still showed green fluorescence, and the red fluorescence was almost negligible. Most of the sonicated cancer cells (96%) also still showed green fluorescence compared to the control group. Similarly, after the acoustic separation of square (94%) and circle (97%) AMPA chips, most of the cancer cells showed high activity. This result indicates that vibration-induced acoustic streaming from microstructures can gently capture cancer cells without causing damage to the cells.

## 4. Discussion

Over the past decades, CTCs have become an important biomarker in liquid biopsies, and monitoring CTC types and quantitative trends can provide valuable assessment and guidance for cancer diagnosis. Currently, various microfluidic platforms have been developed for isolating circulating tumor cancer cells from blood, but developing a low-cost, easy-to-operate, and efficient strategy remains a challenge. In this study, we developed a low-frequency acoustic microstructure array chip (AMPA chip) to systematically investigate the effect of microstructure shape on cancer cell capture efficiency. Using the optimized rhombus micropillar structure, we could achieve precise capture of cancer cells from blood samples with a capture efficiency of up to 93%, which is similar to other reported microfluidic devices used for cancer cell isolation from circulating tumors [38,53]. Therefore, this method is expected to be developed into a clinically useful platform for real-time monitoring of CTC count changes, which will help clinicians adjust the treatment plan in a timely manner and find the optimal time point for treatment. We demonstrate an acoustic-based microfluidic platform with the advantages of label-free, contact-free, highly biocompatible, and no need for sheath flow.

Morphological detection of cancer cells is of key significance in cancer metastasis research. By observing the morphology of CTCs, it is possible to gain insight into their structure and characteristics, thus revealing their metastatic mechanisms and potential effects [41,54]. We have demonstrated that our device is capable of capturing cancer cells at different locations, thus facilitating the observation of cancer cell morphology. Meanwhile, previous studies have shown that rotational manipulation of cells at the capture position can be achieved by this acoustic microfluidic approach, which would provide us with more opportunities to observe cell morphology [39,44]. By analyzing the morphological parameters of CTCs, we can have a more comprehensive understanding of their response and adaptability in different environments, which can provide new ideas and methods for the prevention and treatment of cancer metastasis.

Our device is a gentle platform for cancer cell isolation that maintains high cellular activity, thus making it possible to perform downstream analyses on them, such as cell culture [20] and drug efficacy studies [36]. More importantly, our method also isolates CTC clusters in blood samples. The presence of CTC clusters in the blood of cancer patients may help to understand the metastatic process of human cancers. Studies have shown that CTC clusters can serve as independent prognostic markers for poor patient outcomes [4]. CTC clusters have a shorter half-life in blood and a higher propensity to metastasize compared to individual CTCs [55]. Therefore, further, more in-depth characterization of the role of these clusters in different types of cancers and stages of cancer progression is needed to reveal their potential contribution to the metastatic spread of cancer.

However, our acoustic microfluidic separation device has some limitations. First, we only investigated the effect of microstructure shape on the capture efficiency of cancer cells and did not optimize the design of the microstructure array arrangement. In addition, our device performs separation based on cell size differences. However, the size of cancer cells varies depending on drug treatment and other challenges in the environment, so all types of cancer cells cannot be separated based on size alone [54]. Affinity separation methods do not rely on the physical properties of cancer cells. In this regard, in future work, we hope to combine affinity methods with acoustic separation methods to further improve the applicability of the device.

## 5. Conclusions

In this work, we introduce a novel low-frequency acoustic micropillar array chip (AMPA chip) with optimized micropillar geometry for flexible label-free separation of cancer cells from blood samples. By combining a controllable acoustic field with continuous background flow, the capture of target particles and cancer cells is achieved in the capturing region based solely on their size. Simultaneously, a substantial amount of experimental and simulation results indicates that the rhombus micropillar has a stronger ability to separate cancer cells compared with other microstructures. At a flow rate of 15 µL/min, the rhombus micropillar structure maintains high separation efficiency (93%) as well as cell activity (96%). AMPA chip enables the counting and high activity enrichment of CTCs, demonstrating its promising potential for drug sensitivity testing and formulation of personalized therapies. In addition, this AMPA chip is easy and flexible to operate, and can realize the capture of the same cancer cell at different locations, which is more conducive to optical detection. To further evaluate the potential of the AMPA chip in cancer cell screening technology, extensive clinical validation with a large number of cancer patient samples is required. In conclusion, this novel technology provides an efficient, precise, and highly biocompatible method for the separation of cancer cells, holding promise for positive impacts in biomedical research and cell analysis.

## Figures and Tables

**Figure A1:**
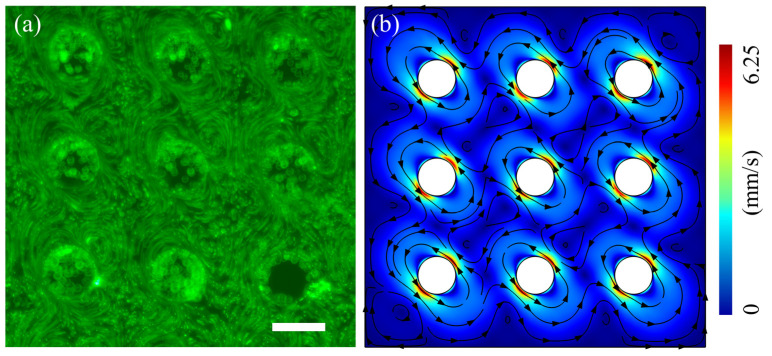
Acoustic streaming results generated by ultrasound-driven parallel microstructures (**a**) Trajectories of fluorescent particles (2 µm) around the corresponding uniformly arranged microstructure arrays, Scale, 100 µm. (**b**) corresponding simulated acoustic streaming results.

## Data Availability

The data that support the findings of this study are available from the corresponding author upon reasonable request.

## Supplementary Materials

The following supporting information can be downloaded at: https://www.mdpi.com/article/10.3390/mi15040421/s1. Video S1: The visualization of Acoustic streaming using fluorescent polystyrene particles with diameters equal to 2 µm; Video S2: The capture and release of 20 µm polystyrene particles by combining Background flows with acoustic field. Video S3: The separation of 5 µm and 20 µm polystyrene particles by the size effect based on the acoustic capturing force. Video S4: The separation of cancer cells by the size effect based on the acoustic capturing force.

## Appendix A. Supplementary of Simulation

### Appendix A.1. Theoretical Analysis

Under external acoustic driving, the liquid equations can be described using the following variables: temperature (*T*), pressure (p), and velocity (v). According to perturbation theory, these variables can be expressed using perturbation series (subscripts 0, 1, 2, respectively) [49]:

(A1) $$T=T_{0}+T_{1}+T_{2}$$

(A2) $$p=p_{0}+p_{1}+p_{2}$$

(A3) $$v=v_{1}+v_{2}$$

where $T_{0}$ and $p_{0}$ are the fixed temperature and pressure before the acoustic wave is present.

The vibration modes of the chip during the actual manipulation are very complex, and an elliptical vibration mode often occurs [42,50]. This situation is described as a microstructure vibration velocity boundary condition of:

(A4a) $$v_{x}=\omega d_{1}e^{-iwt}$$

(A4b) $$v_{y}=i\omega d_{2}e^{iwt}$$

where $v_{x}$ and $v_{y}$ are the vibrational velocities in the x direction and y direction, respectively, $d_{1}$ and $d_{2}$ are the vibrational amplitudes in the direction and direction, respectively, the vibrational amplitude in the x direction is three times the y vibrational amplitude in the direction, and ω is the angular velocity. The micropillar shape boundary is defined as the driving source and the other boundaries are defined as the thermostatic conditions.

Using the thermodynamic heat transfer equation for $T_{1}$, the continuity of motion equation expressed in terms of $p_{1}$, and the kinetic Navier–Stokes equation for $v_{1}$, the first order control equation can be expressed as:

(A5) $$\frac{\partial T_{1}}{\partial t}=D_{th}\nabla^{2}T_{1}+\frac{\alpha T_{0}}{\rho_{0}C_{p}}\frac{\partial p_{1}}{\partial t}$$

(A6) $$\frac{\partial p_{1}}{\partial t}=\frac{1}{\gamma \kappa_{0}}\left[\alpha \frac{\partial T_{1}}{\partial t}-\nabla \cdot v_{1}\right]$$

(A7) $$\rho_{0}\frac{\partial v_{1}}{\partial t}=-\nabla p_{1}+\eta \nabla^{2}v_{1}+\beta \eta \nabla \left(\nabla \cdot v_{1}\right)$$

where $D_{th}$ is the thermal diffusivity, $C_{p}$ is the specific heat capacity, α is the isobaric thermal expansion coefficient, $\kappa_{0}$ is the isentropic compressibility, γ is the specific heat capacity ratio, η is the dynamic viscosity, and β is the viscosity ratio.

For water and most liquids, the thermal effects in the first-order equations are very small, so they are neglected to simplify the subsequent analysis. In previous studies, microsecond oscillations could not be resolved [48,49]. Therefore, we utilize the time averaged over the full oscillation period (denoted as 〈⋅⋅⋅〉) to describe the second-order continuity equation and the Navier-Stokes equation:

(A8) $$\rho_{0}\nabla \cdot \langle v_{2}\rangle =-\nabla \cdot \langle \rho_{1}v_{1}\rangle$$

(A9) $$\eta \nabla^{2}\langle v_{2}\rangle +\beta \eta \nabla \left(\nabla \cdot \langle v_{2}\rangle \right)-\langle \nabla p_{2}\rangle =\langle \rho_{1}\frac{\partial v_{1}}{\partial t}\rangle +\rho_{0}\langle \left(v_{1}\cdot \nabla \right)v_{1}\rangle$$

It can be seen that the left second order field can be expressed as a product of the right first order field.

### Appendix A.2. Model Procedures and Parameters

Simulation results of the acoustic streaming were obtained using the finite element analysis software COMSOL Multiphysics 5.5. The simulation domain was restricted to a square box (500 µm × 500 µm). The microstructures are defined as three shapes of micropillars (circle, rhombus, and square) with feature size a=100 µm, respectively, located in the middle of the square box. The boundaries of the three micropillars shapes were defined as the driving source and the other surfaces were defined as anti-slip boundaries. The amplitude of the driving source in the x direction is set to 50 µm, and the amplitude in the y direction is set to 150 µm. After setting up the boundary conditions for the vibration velocity of the micropillar according to Equations (A4a) and (A4b), the acoustic pressure field and the first order velocity can be solved through the thermoacoustic physics interface. Then, we converted the first order velocity into a mass source term and a volume force term, and calculated the time-averaged second order acoustic streaming <v2> using the laminar physics interface. The specific parameters used in the simulation process are shown in Table A1.

**Table A1:** Parameters used in the simulation and their values.

Parameter	Value	Unit
Fluid density, $\rho_{0}$	997	kgm^−3^
Sound speed of the fluid, $c_{0}$	1496.73	m^−3^
Fluid compressibility, $\kappa_{0}$	4.45 × 10^−10^	Pa^−1^
isobaric Thermal expansion coefficient, α	2.57 × 10^−4^	K^−1^
Specific heat capacity, $C_{p}$	4181.5	Jkg^−1^K^−1^
Thermal diffusivity, $D_{th}$	1.43 × 10^−7^	m^2^s^−1^
Specific heat capacity ratio, γ	1.0106	1
viscosity ratio, β	1/3	1
Dynamic viscosity, η	8.9 × 10^−4^	Pas
Forcing frequency, f	45	kHz
Vibration amplitude in x direction, $d_{1}$	50	nm
Vibration amplitude in x direction, $d_{2}$	150	nm

## Appendix B

**Table A2:** Substances in blood samples [51].

Ingredient	Concentration (mL^−1^)	Diameter (µm)	Shape
White blood cell	(4–10) × 10^6^	10–12	sphere
Red blood cell	(3.5–5.5) × 10^9^	7–8	sphere
Blood platelet	(2–5) × 10^8^	2–3	Biconvex
Plasma	(-)	(-)	(-)
